# Supplementary material for: Integrated neural dynamics of sensorimotor decisions and actions
Source: PLoS Biol. 2022 Dec 15;20(12):e3001861. doi: 10.1371/journal.pbio.3001861 (PMC9754259; doi:10.1371/journal.pbio.3001861)
Supplement: S1 Text — Fig A. Schematic diagram of the analysis approach. Step 1: Neural activity is recorded from individual cells while the monkey performs individual trials of different types (e.g., “easy trial to the right,” “misleading trial to the left,” etc.), and the temporal profile is aligned on movement onset time. Step 2: For each cell, activity is averaged from all trials during the slow block in which the target on the right was chosen, to produce an average “Slow-R all” temporal profile. This is repeated for Slow-L, Fast-R, and Fast-L trial groups. Step 3: The four trial groups are concatenated into a single row for each cell, and the rows of all cells grouped into a large matrix. Step 4: Principal components analysis is performed on that matrix to produce the “loading matrix” L. Step 5: To generate the trajectory for a specific trial type and a specific population, we first average the neural activity of a given cell over all trials of the given type (e.g., misleading trials in the slow block in which the right target was chosen). Step 6: To compute the profile for that trial type and for a given principal component, the average activity of each cell is weighted by the corresponding row (cell) and column (PC) of the loading matrix L and summed. Step 7: The profile of each PC is divided by the number of cells used. Step 8: The resulting PC profiles are plotted against each other to produce neural trajectories. Here, the trajectory for Slow-Misleading-R trials is highlighted and superimposed over the trajectories of other slow block trials, as in Fig 4a. Note that steps 5–8 can be computed using arbitrary subsets of data (different trial groupings, different subsets of cells), and arbitrary alignment (on movement onset or on the first token jump), because all they depend upon is the loading matrix computed by steps 1–4. Fig B. The top four principal components produced when the loading matrix was computed from each brain region separately, same format as Fig 2. On the right [file pbio.3001861.s001.pdf]

Integrated neural dynamics of sensorimotor decisions and actions

David Thura, Jean-François Cabana, Albert Feghaly, and Paul Cisek

Groupe de recherche sur la signalisation neurale et la circuiterie
Department of Neuroscience
Université de Montréal
Montréal, Québec, CANADA

Supplemental information

Robustness of results using different approaches for computing principal components

There are several possible approaches for computing the principal components of neural activity, each with strengths and weaknesses. For the analyses shown in the main body of this paper, we followed the approach described in the Methods and outlined below in Fig A. This can be thought of as two separate procedures: one to produce the loading matrix L (steps 1-4), and one to use that matrix to calculate the profiles of principal components for given trial types and neurons (steps 5-8). Note that computing the loading matrix is done by grouping trials into only four large trial classes (Slow-Right, Slow-Left, Fast-Right, Fast-Left), but once the matrix has been computed, it can be used to calculate the profiles of principal components for arbitrary groupings of trial types or cells, and for arbitrary epochs of time aligned to different events. For example, the temporal profile of PCs from dlPFC can be computed as a weighted average of activity from those cells using only the rows of the loading matrix corresponding to dlPFC cells.

With this approach, the PCs have a consistent relationship across different brain regions: e.g., PC1 is always related to commitment, while PC2 captures evidence and directional tuning (in regions that possess such sensitivity). However, it is also possible to perform the analysis separately on cells within each region. The result is shown in Fig B, for each of the five regions in which we recorded. While the ordering of components now differs from what was shown in Figs 2 and 5, the same basic shapes are again observed. In particular, as in Fig 5, PMd and M1 still exhibit the same four top PCs (albeit PC3 and PC4 are switched in M1), while dlPFC primarily shows a component related to sensory evidence, which now becomes PC1, correlates with evidence with R=0.94, and explains 42.8% of the variance. The top four PCs for GPe and GPi are also remarkably similar to those obtained when the PCA was performed on all cells together.

Fig C shows additional approaches for computing the loading matrix: A) Using the same number of neurons from each population (35 because that was the smallest count of cells recorded in both Slow and Fast blocks, in GPe); B) Computing L only using data after movement onset; C) Leaving out the square root transform of neural firing rates; D) Using only data from the Slow block; E) Providing the PCA with data from all 28 conditions rather than the four used in the main analyses. As can be clearly seen, in nearly all cases the component profiles constructed using the loading matrix exhibit the same features. There is always a component related to commitment (PC1 except for panel B), and there is always a component that is well correlated (R^2>0.9) to the profile of sensory evidence during deliberation, *even* when only movement time data was used to compute L (panel B). Not surprisingly, if only Slow block data is used to compute L (panel D) then there is no component showing the block-dependent difference, but the others are still present.

Fig D repeats our analyses separately for data from the two different monkeys used in our experiments. Again, the same components are found, albeit PC1 and PC2 are switched (compare panels A and B). Furthermore, the differences in properties across the five regions are still the same (panels C and D) and the shapes of the manifolds are still consistent: curved in PMd and flat in M1 (panels E and F).

Finally, Fig E repeats our analyses without using the cell duplication step described in the Methods. Once again we see the same general features. The top four PCs are found once again (panel A), and although they are no longer perfectly invariant or symmetric with respect to direction, they still afford the same functional interpretations: PC1 reflects commitment, PC2 correlates with evidence (R^2=0.92), PC3 reflects the block type, and PC4 reflects elapsing time. Furthermore, the decision manifold of PMd is still curved (see panel B and the inset) while that of M1 is flat (panel C), and the properties of dlPFC, GPe, and GPi resemble those shown in Fig 7. The structure of the loading matrixes is not more difficult to discern, but the orthogonality of dlPFC and GPi is still clear.

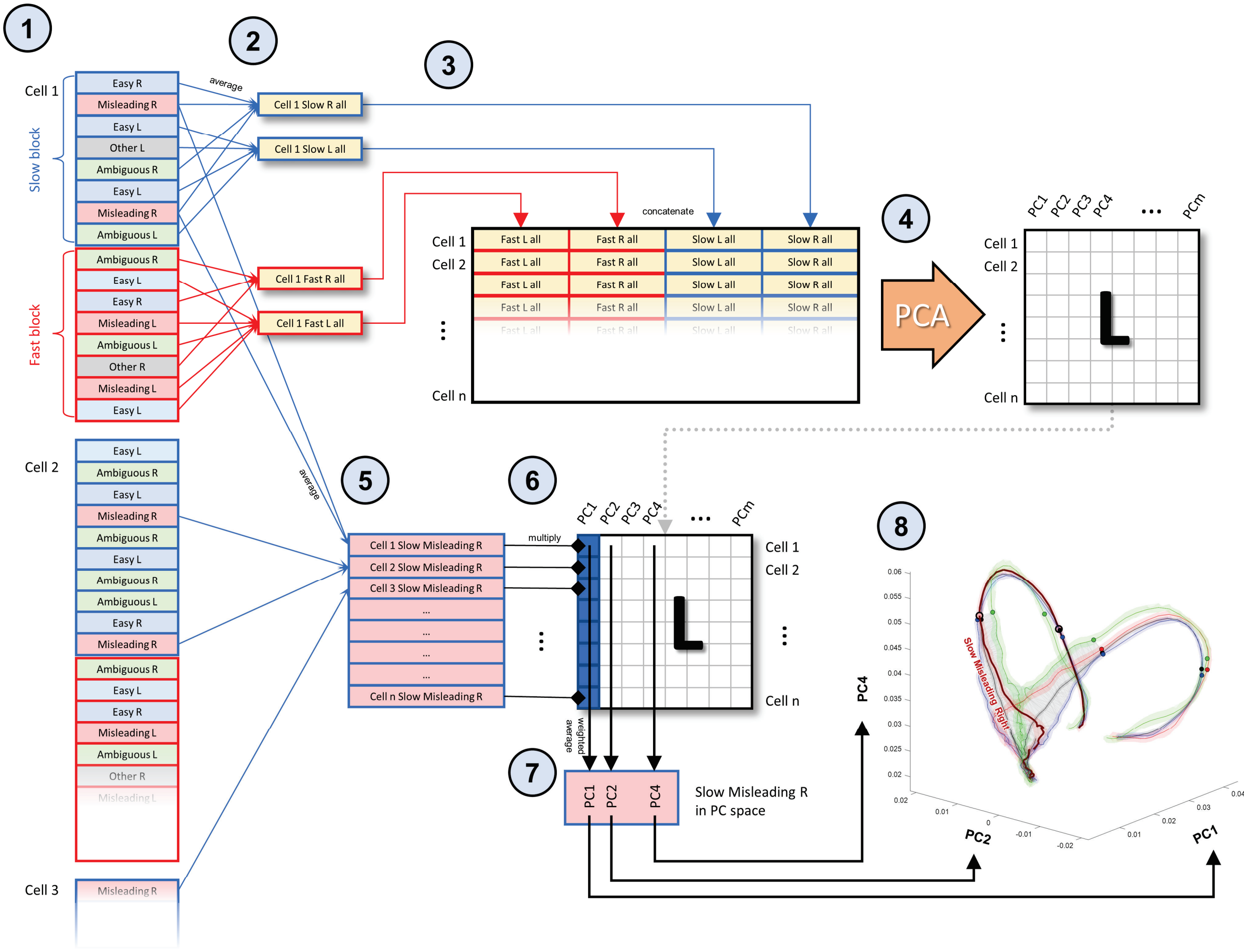

Fig A. Schematic diagram of the analysis approach. Step 1: Neural activity is recorded from individual cells while the monkey performs individual trials of different types (e.g., “easy trial to the right”, “misleading trial to the left”, etc.), and the temporal profile is aligned on movement onset time. Step 2: For each cell, activity is averaged from all trials during the slow block in which the target on the right was chosen, to produce an average “Slow-R all” temporal profile. This is repeated for Slow-L, Fast-R, and Fast-L trial groups. Step 3: The four trial groups are concatenated into a single row for each cell, and the rows of all cells grouped into a large matrix. Step 4: Principal components analysis is performed on that matrix to produce the “loading matrix” L. Step 5: To generate the trajectory for a specific trial type and a specific population, we first average the neural activity of a given cell over all trials of the given type (e.g. misleading trials in the slow block in which the right target was chosen). Step 6: To compute the profile for that trial type and for a given principal component, the average activity of each cell is weighted by the corresponding row (cell) and column (PC) of the loading matrix L, and summed. Step 7: The profile of each PC is divided by the number of cells used. Step 8: The resulting PC profiles are plotted against each other to produce neural trajectories. Here, the trajectory for Slow-Misleading-R trials is highlighted and superimposed over the trajectories of other slow block trials, as in Fig 4A. Note that steps 5-8 can be computed using arbitrary subsets of data (different trial groupings, different subsets of cells), and arbitrary alignment (on movement onset or on the first token jump), because all they depend upon is the loading matrix computed by steps 1-4.

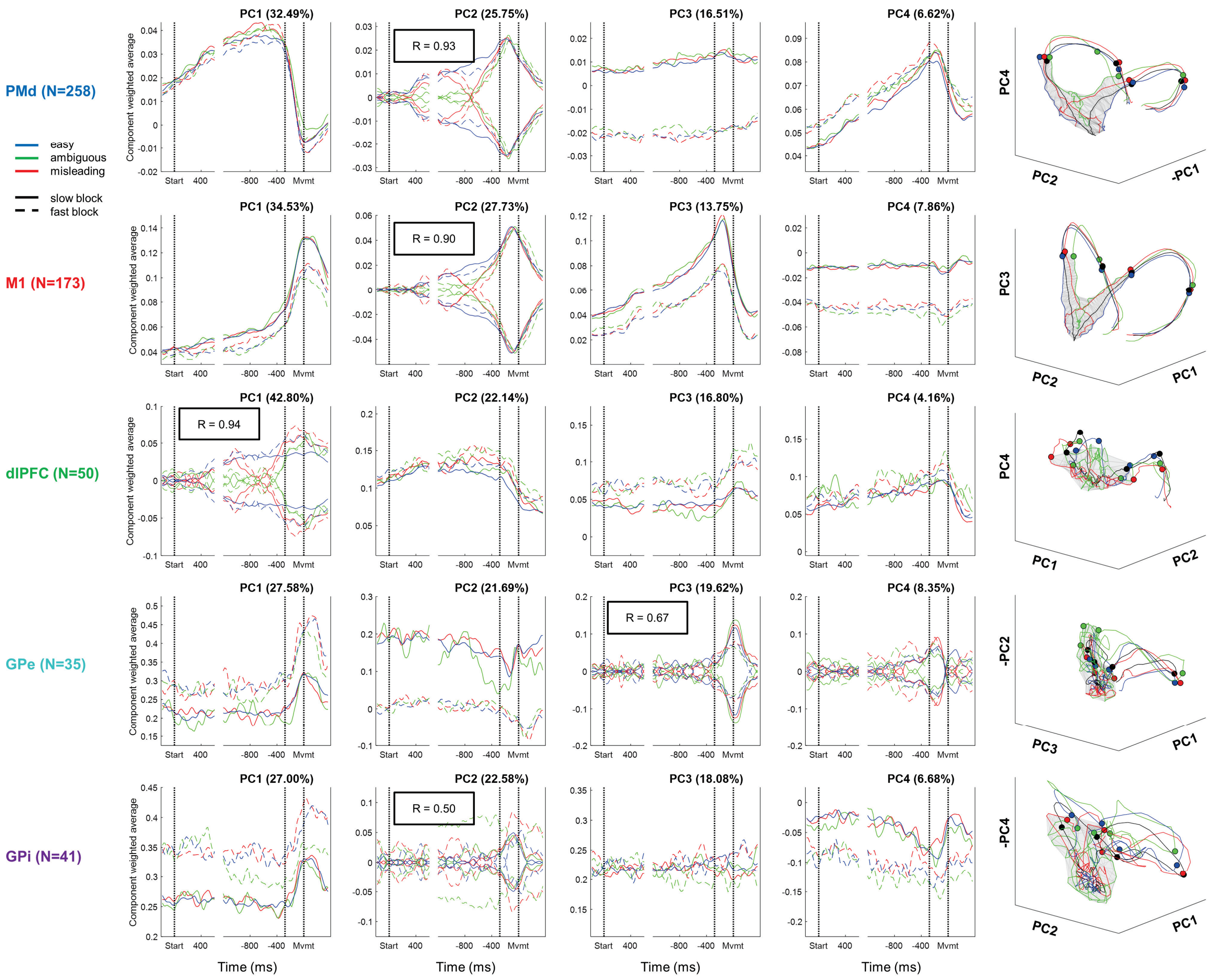

Fig B. The top four principal components produced when the loading matrix was computed from each brain region separately, same format as Fig 2. On the right are shown the neural trajectories computed in the space of the components that reflect commitment, evidence, and elapsing time. Note that in some cases the axis is inverted to facilitate comparison of these neural spaces to those shown in Figs 6 and 7. Data and code available at https://doi.org/10.6084/m9.figshare.20805586.

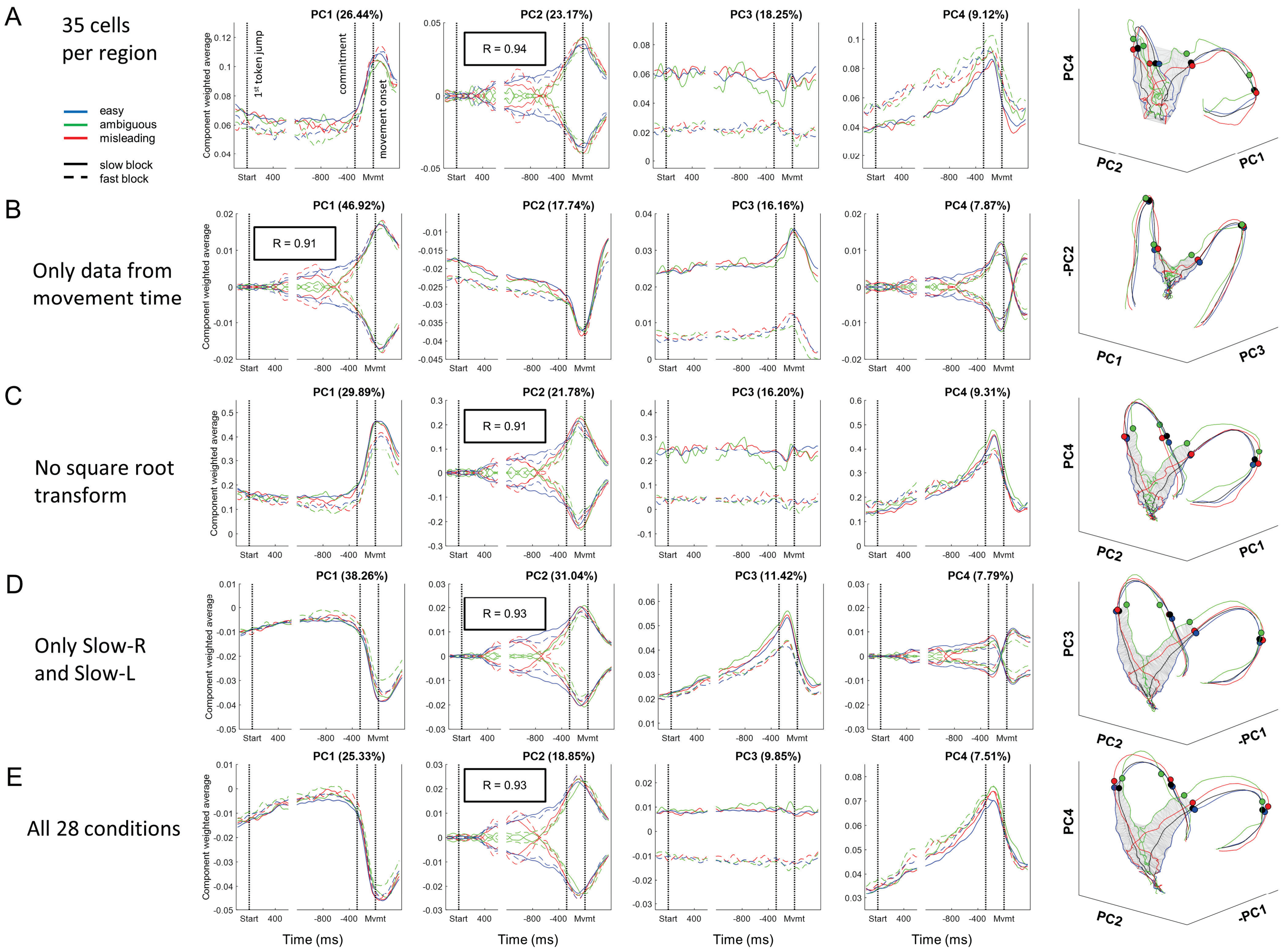

Fig C. The top four principal components produced when the loading matrix was computed using only 35 cells per region (A), only data from movement onset to 400ms later (B), data without square root transformation (C), only data from the slow block (D), or all data from all 28 trial conditions separately (E). Same format as Fig B. Data and code available at https://doi.org/10.6084/m9.figshare.20805586.

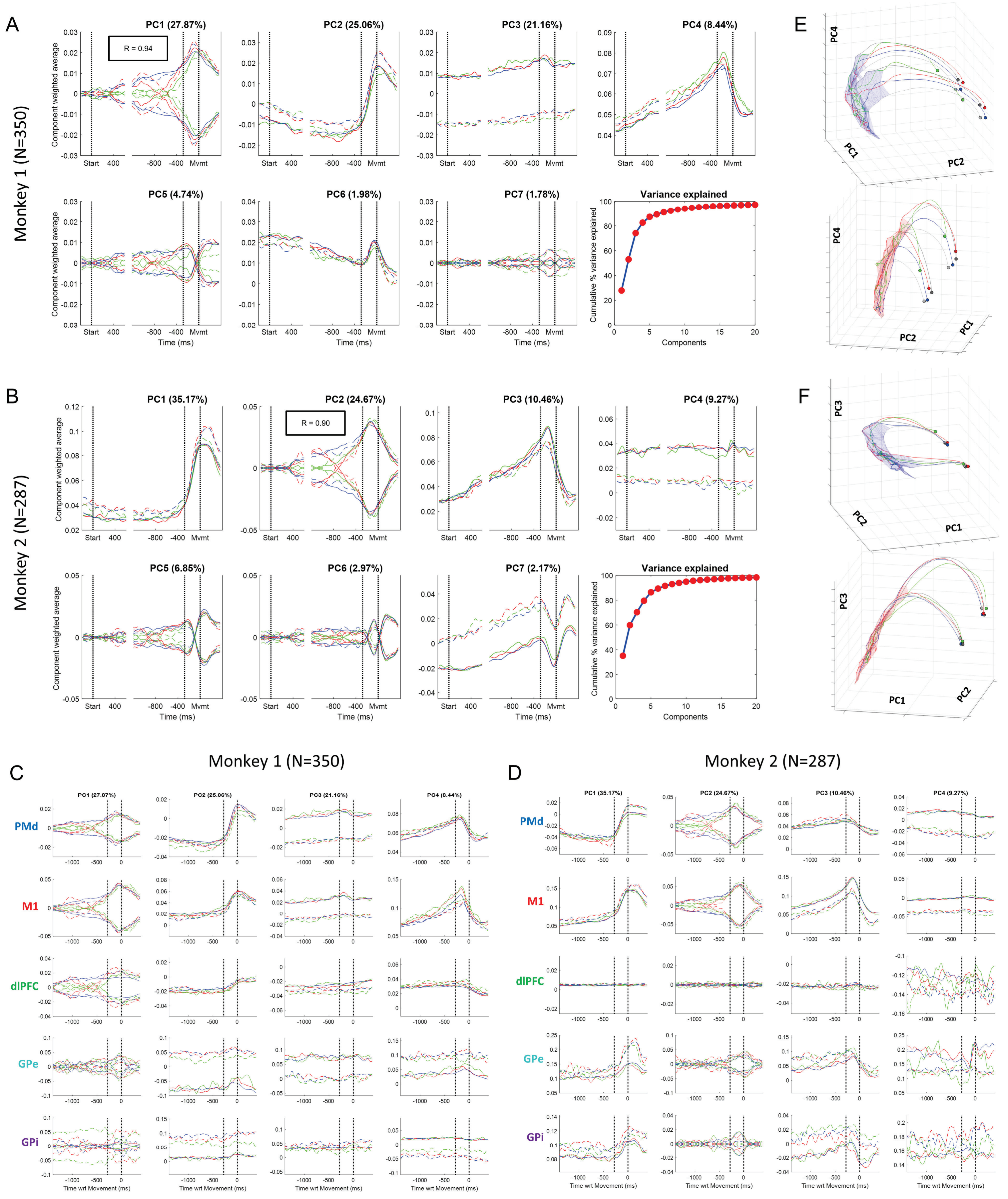

Fig D. Results computed separately for each of the two animals. Panels A and B show the principal components (same format as Fig 2). Panels C and D show the top four components calculated using each brain region (similar to Fig 5). Panels E and F show the decision manifolds for PMd and M1 (similar to Fig 6). Data and code available at https://doi.org/10.6084/m9.figshare.20805586.

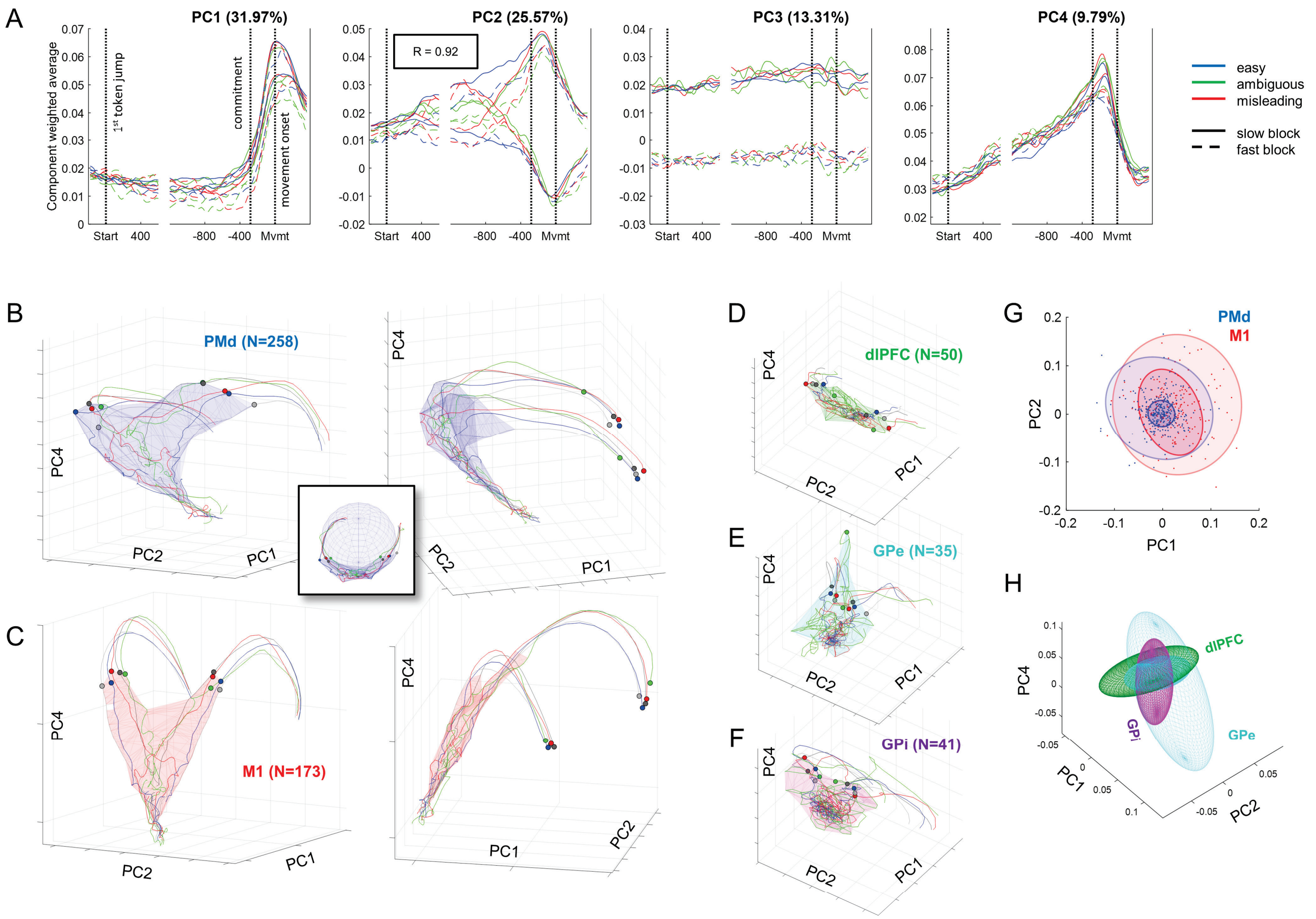

Fig E. Results computed without the cell duplication step. A. The top four principal components (similar to Fig 2). B. The neural trajectories and decision manifold for PMd (similar to Fig 6A). C. Neural trajectories and decision manifold for M1 (similar to Fig 2B). D-F. Neural trajectories for dlPFC, GPe, and GPi (similar to Fig 7). G. Loading distributions for PMd and M1 with respect to PC1 and PC2 (similar to Fig 8A and 8C, left). H. Loading distributions for dlPFC, GPe, and GPi with respect to PC1, PC2, and PC4 (similar to Fig 8F). Data and code available at https://doi.org/10.6084/m9.figshare.20805586.

Distinguishing gradients versus clusters of cell properties

One of our key results is the apparent absence of distinct neural categories in our populations (Fig 8), arguing for a unified dynamical system of cells with continuous properties. However, is this continuity real or could it be an artifact of dimensionality reduction? To address this question, we created a variety of synthetic populations of neuron-like units, in which we deliberately created specific categories, and then applied to them the same analyses we used to examine real data. If our analyses fail to find these categories, then we cannot be confident that real categories do not exist in the real data. We attempted a variety of different ways to construct synthetic populations, but here report on two, each of which constructs synthetic units using combinations of the PCs we obtained from the real data (Fig 2).

The first population consisted of three distinct groups of cells. Group 1 consisted of 200 neurons with loading coefficients of 1 on PC1 and PC2, and zero for all others. Group 2 consisted of 200 neurons with loading of 1 on PC2 and PC4 and zero for all others. Group 3 consisted of 200 neurons with loading of 1 on PC1 and PC4 and zero on others. These loading coefficients were multiplied by $1+0.25N$, where N is a normally distributed random variable with mean 0 and standard deviation 1. The synthetic activity of each cell was then computed as a sum of the temporal profile of each PC weighted by its loading coefficient for that cell. Finally, noise was added by multiplying the value of each bin of activity by a uniformly distributed random variable between 0 and 1.

We then subjected these synthetic units to exactly the same analyses we used for our real data, including square-root transformation, smoothing, and duplication. PCA analysis was then used to compute loading matrices of "synthetic" principal components (SPCs). The temporal profiles of the SPCs were then computed using a weighted sum of all units, and the loading matrix was analyzed using GMMs. For the synthetic data, we used 4-dimensional Gaussians to analyze the loading matrix.

As shown in Fig Fa, the "synthetic" principal components (SPCs) almost perfectly capture the original PCs from which the cells were built, albeit in a different order (SPC1 is like PC2, SPC2 is like PC1, and SPC3 is like PC4). Furthermore, although the GMM analysis was applied to all 600 units together, without information on how the different groups were built, it correctly identified the relevant clusters (Fig Fb).

The second synthetic population consisted of 600 neurons that all had loading coefficients of 1 on PC1 and PC2 and zero for others, again multiplied by $1+0.25N$, turned into activity profiles using a weighted sum of the real PCs 1 and 2, and multiplied by a random variable uniformly distributed between 0 and 1. Next, these 600 units were split into three groups: For "deliberation" units, activity before commitment was multiplied by 1, activity between commitment and movement was scaled by a number linearly dropping from 1 to zero, and activity after movement onset was set to zero. For "movement" units, activity before commitment was set to zero, activity between commitment and movement was scaled by a number linearly rising from zero to 1, and activity after movement onset was multiplied by 1. For "mixed" units, all activity was kept unchanged.

We then ran PCA and obtained the synthetic components shown in Fig Fc. Note that SPC1 is similar to PC1, SPC2 is like PC2, but now we also find an SPC3 that looks like PC2 except for a switch of activity between deliberation and movement. This is reminiscent of some of the higher components we found in the neural data (see PC5 and PC6 in Fig 2). Importantly, *no such component was used to build the synthetic units*. So where did it come from? The answer lies in the PCA algorithm, which sequentially identifies components on the basis of variance explained. After finding SPC1 it discovered SPC2, which together explain a large proportion of activity for both "deliberation" and "mixed" units. Since no linear combination of these two components could explain "movement" units, the next component (SPC3) captures some of the remaining unaccounted variance. In particular, note that a linear combination of SPC2 plus 3 times SPC3 can cancel out deliberation activity and produce a "movement" unit.

These observations on synthetic data suggest that the higher principal components we found in our real data (PCs 5, 6, 7, etc.) also do not necessarily reveal additional “higher order” features of neural dynamics, but simply result from the type of heterogeneity that has long been observed in sensorimotor cortical regions (1-6). But this then revives the question of functional clusters of real neurons – might distinct categories exist in these regions? If they did, would our analysis of loading matrices identify them correctly? Fig Fd shows the result of GMM fits to the entire population of 600 synthetic units, again performed without any information on the underlying groups. Clearly, the relevant clusters were found. In particular, “movement” units (red) were identified with two Gaussians (one for right-tuned and one for left-tuned units), loaded positively onto SPC1, and onto both SPC2 and SPC3 with 3 times larger coefficients for the latter – consequently cancelling out their deliberation-time activity. In contrast, “deliberation” units (blue) were identified with two oriented Gaussians orthogonal to those of “movement” units and negatively loaded on SPC1. Finally, “mixed” units (green) were identified as clusters lying in-between. This demonstrates that if distinct categories of neurons really did exist in the real populations we recorded, the GMM analysis would have identified them correctly.

An alternative strategy that tests for the presence of distinct neural categories is to compute the angular direction of the vector of loading coefficients of each neuron, and then examine whether the distribution of pairwise angular differences between these vectors deviates outside of what would be expected from a spherically uniform population. This has been called the “projection angle index of response similarity” (PAIRS) test (7) and modified to compare against an elliptical uniform population (ePAIRS) test (8). However, such strategies test against the null hypothesis of uniformity, and do not distinguish between non-uniform distributions with separate clusters versus non-uniform continuous distributions without clusters. We can already clearly see that our distributions are non-uniform – for instance, PMd tends to be positively loaded onto PC4 (Fig 8A, rightmost plot) while dlPFC tends to be negatively loaded onto PC1 (Fig 8E, leftmost plot). Thus, PAIRS or ePAIRS tests will (correctly) reject their null hypothesis but not inform us of whether or not clusters exist in the population (as in Fig F). The GMM approach is therefore better suited for addressing the specific question of whether our populations contain clusters, and for identifying what those clusters are.

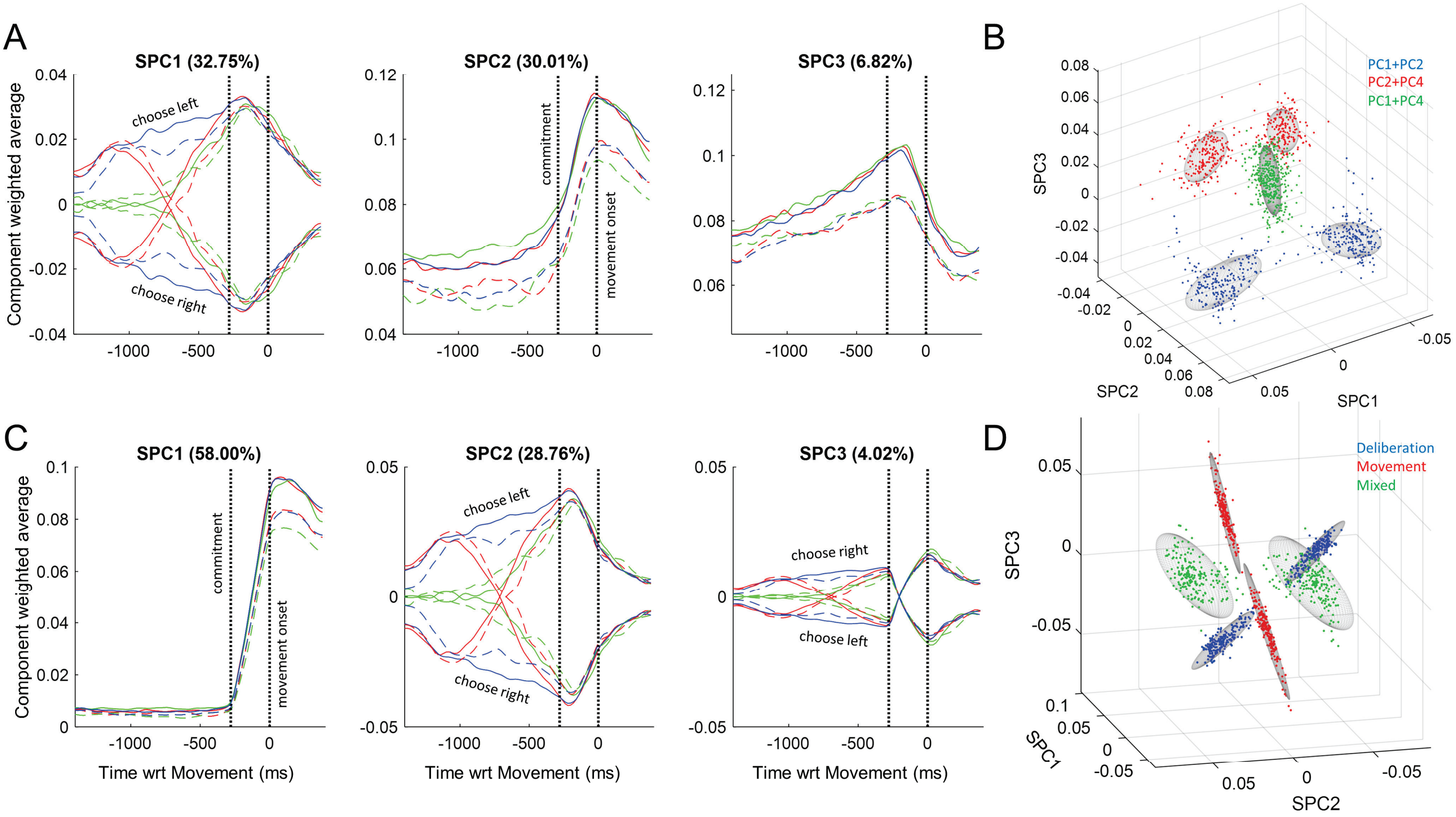

Fig F. Analyses of synthetic populations. A. The top 3 Synthetic PCs (SPCs) obtained from a population of 600 neurons constructed using combinations of the original PCs 1, 2, and 4 (see Fig 2). B. The loading coefficients of all 600 neurons, fitted with GMMs (gray ellipsoids). Colors indicate the three groups of neurons built with different combinations of those original PCs (see legend). C. The top 3 SPCs obtained from a population of 600 neurons constructed only using the original PC1 and PC2, but separated into three distinct categories: cells that are only active during deliberation, cells only active during movement, and cells active during both epochs. D. The loading coefficients of all 600 neurons, fitted with GMMs (gray ellipsoids). Colors indicate the three categories of neurons. Data and code available at https://doi.org/10.6084/m9.figshare.20805586.

Interpreting the shapes of decision manifolds

One of the most striking findings of our study is that the state of neural activity during deliberation appears to be confined to a thin manifold that possesses robustly different shapes in different regions. Most interesting is the difference between a quasi-spherically curved manifold in PMd and a remarkably flat manifold in M1. We believe that these shapes, consistently observed regardless of how we perform the analysis, reveal a fundamental difference between the dynamics of the two regions. While determining those dynamics is admittedly challenging given our current data, here we suggest two plausible interpretations inspired by previous work on computational models of recurrent neural circuits (9-12).

First, we consider why the PMd manifold should curve as if it lies upon a surface of a sphere, while the M1 manifold is nearly perfectly flat. One possible explanation is related to the dynamics of recurrent competitive attractor networks. We illustrate this using a very simple system consisting of two neurons that compete against each other through recurrent inhibition. Note that this minimal model is not intended to simulate our data, but simply to demonstrate the possibility that some features of our data (e.g. shape of the decision manifold) could be the result of very general properties of recurrent non-linear dynamical systems.

Let's consider two neurons whose activity is denoted as x_1 and x_2 (Fig Ga) and governed by the following differential equation (9)

$$\frac{dx_i}{dt} = (1 - x_i)\big(E_i + U + f(x_i)\big) - x_i\left(A + f\big(x_j\big)\right) \qquad \text{(S1)}$$

On the right-hand side of equation (S1), the first term is excitation and the second is inhibition. E_i is the input evidence for choice i, U is the urgency signal, A is a passive decay rate, $f(x_i)$ is the recurrent excitation of each cell to itself, and $f(x_j)$ is the recurrent inhibition from the other cell, j. Note that the terms $(1 - x_i)$ and $-x_i$ in equation (S1) ensure that cell activities are always in the interval from 0 to 1. The function $f(x)$ is sigmoidal of the form

$$f(x) = \frac{G}{1+e^{-S(x-0.7)}} \qquad \text{(S2)}$$

where G is the gain and S is the steepness of the sigmoid. Fig G schematizes this simple model and illustrates some of its dynamics. Panel B shows the flow field of the system in the (x_1, x_2)-plane assuming a function $f(x)$ with high gain and a steep slope, when the evidence is balanced. Note how the flow field quickly pushes the neural state to a central stable region, outlined in purple, within which the flow is slower. In the presence of noise, the system will be strongly constrained to remain inside this region, but can shift within it relatively easily. As the balance of evidence changes, the stable region shifts in the plane (Panel C) but always lies oriented along that straight line. As a result, activity is normalized such that x_1+x_2 is approximately constant (L1-normalization). In contrast, panels D and E show a system where the interaction function has low gain and a shallow slope. Note that the stable region for different input patterns still shifts in the (x_1, x_2)-plane, but not along a straight line (panel E). Instead, the different stable regions now lie along a circular path, such that $x_1^2 + x_2^2$ is approximately constant (L2-normalization). In both systems, the state is strongly confined into a narrow subregion of the full space (a "decision manifold"), but can shift within it due to changes of evidence as well as noise. If the value of U is increased, this subspace will shift toward the upper right (not shown) until the system bifurcates into two stable attractors, each corresponding to choosing either x_1 or x_2. This simple model proposes a straightforward candidate explanation for the difference in shapes of the decision manifolds in PMd versus M1. Note that in the simulations shown here, the input itself is L1-normalized, such that $E_1 + E_2$ is constant. However, similar shapes emerge if the input is instead L2-normalized (such that $E_1^2 + E_2^2$ is constant); i.e. the sharp sigmoid still produces a flat manifold while the gentle sigmoid still produces a curved one (Fig Gf). In other words, the shapes

shown in panels C and E result primarily from properties of the network dynamics, not properties of the input.

In addition to its curvature in the PC1-PC2 plane, the PMd manifold also exhibits a curvature in the PC1-PC4 plane. In particular, it initially leans in the negative PC1 direction and then bends toward the positive PC1 direction prior to commitment (see Fig 6A, right). This phenomenon could potentially be caused by an inhibitory influence, which prevents premature movement before selection is complete (13) by keeping PMd away from commitment, but which is gradually overcome by positive feedback in the recurrent circuit between PMd and GPi (12, 14, 15). According to this hypothesis, as the cortical activity becomes increasingly biased in favor of one target over another, it gradually begins to produce the emergence of choice selectivity in the GPe, about 200ms before commitment (see Fig 5, row 4, PC2). When that becomes strong enough to engage selectivity in the GPi, it in turn strengthens emerging selectivity in the thalamus, which then further strengthens selectivity in the cortex. Thus, a positive feedback is established leading to a winner-take-all process that overcomes inhibition and constitutes volitional commitment. This hypothesis predicts a relationship between how tuning emerges in GPi and how and when the PMd state begins to flow toward commitment.

To test this prediction, we examined the correlation between the flow toward commitment in PMd (as reflected in the rate of change of PC1 during deliberation) with the depth of directional selectivity in GPi (as reflected in the absolute value of PC2). Fig Ha shows this comparison for 6 large trial groups: all rightward choices in Slow and Fast blocks, early rightward choices in Slow and Fast blocks, and late rightward choices in Slow and Fast blocks (see Methods for trial definitions). Leftward decision trials were not considered because they are symmetric with respect to rightward decision trials and thus redundant. As can be seen for the different trial types, except for a constant scaling factor the match between these two very different variables is strong, particularly in the epoch between commitment and movement onset. Even if the data is restricted to the period *before* commitment, the correlation for each trial group shown in the individual panels of Fig Ha is significant at $p<10^{-10}$ with Pearson's R above 0.86, and for all trials together with R=0.84 (Fig Hb). Furthermore, across these six conditions there was a significant correlation (p=0.0029, R=0.9556) between the time when the PMd manifold began to tilt toward commitment (derivative of PC1 became consistently positive) and the time when tuning became significant in GPi (95% CI of PC2 no longer included 0). Similar results were not obtained when comparing GPi PC2 against the derivative of PC1 computed from other regions (although there was a weaker but significant correlation with the derivative of PC1 in M1). It is important to note that this particular prediction – a relationship between the derivative of one component in PMd and the absolute value of another in GPi – is not arbitrary. It is motivated by the specific proposal that the timing of the bend in the PMd decision manifold in the PC1-PC4 plane (5a, right) is related to the timing of how tuning emerges in the basal ganglia, which is itself motivated by the hypothesis that both of these variables reflect positive feedback in a recurrent attractor circuit. Of course, like any correlation analysis, it cannot conclusively prove a specific causal relationship. An alternative, though not mutually exclusive hypothesis is that both of these regions are influenced by a common source of inhibition that is gradually released as commitment approaches.

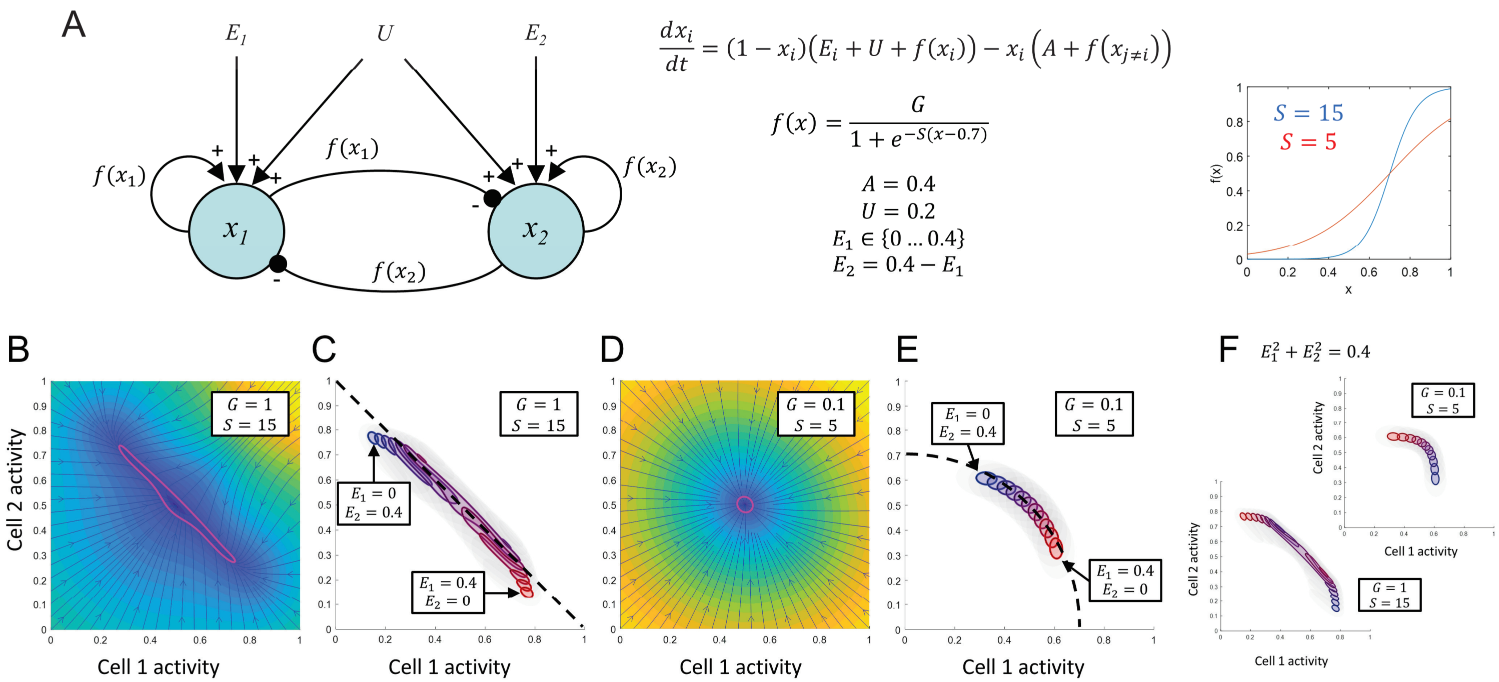

Fig G. Simulation of a simple 2-neuron recurrent competitive attractor. A. The structure of the model is shown at left, next to the governing equations and parameter settings. Two forms of the interaction function f(x) are shown in the inset at right. B. The flow field for a system with high gain (G=1) and steep slope (S=15) when the input evidence is balanced such that E_1=E_2=0.2. Blue arrows depict the flow and shading indicates the speed (yellow=fastest, dark blue=slowest). The purple outline indicates a region around the stable equilibrium, in which the state will tend to remain even with substantial noise. Note that the dynamics flow quickly toward that region and then move more slowly within it. C. The stable regions of the same system for several input patterns, ranging from strong evidence in favor of choice 2 (top left, blue) to strong evidence in favor of choice 1 (bottom right, red). Note that these align in what is approximately a straight line across the state space (compare to dashed black line). D. Same as B but for a system in which the interaction function has low gain (G=0.1) and a shallow slope (S=5). E. Stable regions of the system in D. Note that now, the stable regions for the same range of input patterns do not form a straight line but rather fall on a curve roughly equivalent to a circle (dashed black curve). F. Same as C and E but for models in which the input itself is constrained to lie on a curve.

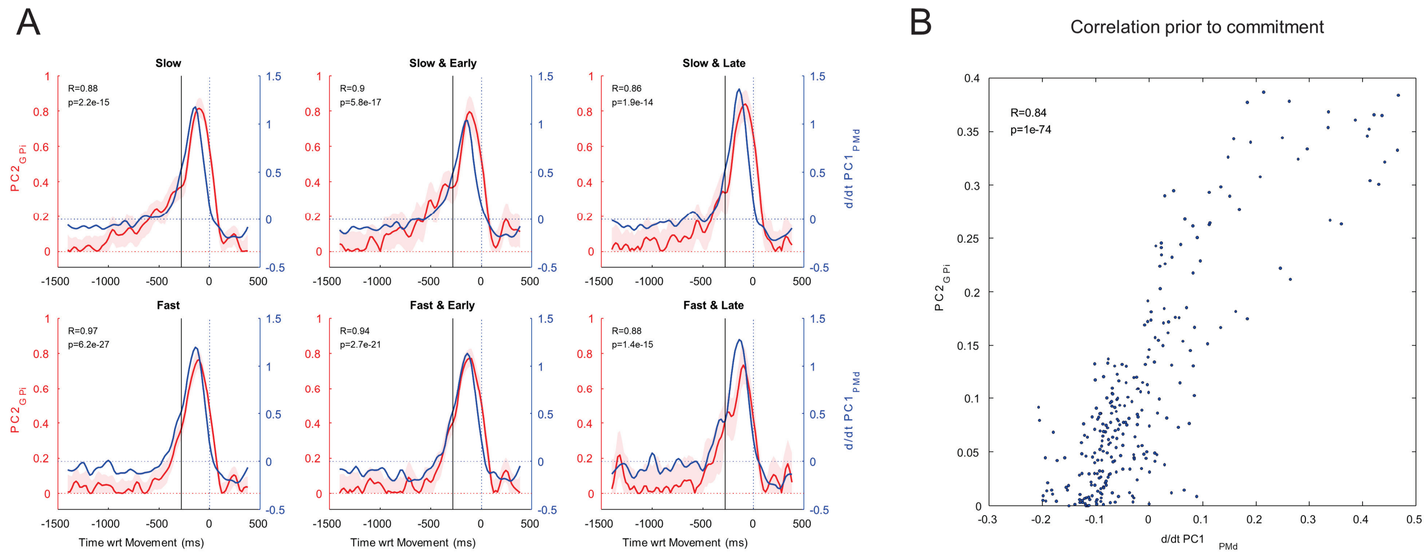

Fig H. Comparison of selectivity in GPi with the slope of the PMd decision manifold. A. Comparison within six conditions: all Slow block trials, Slow block trials where DT<1400, Slow block trials where DT>1400, all Fast block trials, Fast block trials where DT<950, Fast block trials where DT>950. Each panel plots the absolute value of PC2 computed from GPi (red, left y-axis, shaded region indicates 95% CI) along with the derivative of PC1 computed from PMd (blue, right y-axis). The black vertical line indicates the estimated time of commitment. At the top left corner are Pearson's R and the p-value of the correlation between these two signals from 1000ms to 280ms before movement. B. The correlation of these signals, from 1000 to 280ms before movement onset, across all 6 conditions. Data and code available at https://doi.org/10.6084/m9.figshare.20805586.

References

1. Kalaska JF, Cohen DAD, Hyde ML, Prud'homme MJ. A comparison of movement direction-related versus load direction-related activity in primate motor cortex, using a two-dimensional reaching task. Journal of Neuroscience. 1989;9(6):2080-102.

2. Crammond DJ, Kalaska JF. Prior information in motor and premotor cortex: activity during the delay period and effect on pre-movement activity. Journal of Neurophysiology. 2000;84(2):986-1005.

3. Wallis JD, Miller EK. From rule to response: neuronal processes in the premotor and prefrontal cortex. Journal of Neurophysiology. 2003;90(3):1790-806.

4. Cisek P, Kalaska JF. Neural correlates of reaching decisions in dorsal premotor cortex: Specification of multiple direction choices and final selection of action. Neuron. 2005;45(5):801-14.

5. Churchland MM, Shenoy KV. Temporal complexity and heterogeneity of single-neuron activity in premotor and motor cortex. J Neurophysiol. 2007;97(6):4235-57.

6. Hoshi E, Tanji J. Distinctions between dorsal and ventral premotor areas: anatomical connectivity and functional properties. Current Opinion in Neurobiology. 2007;17(2):234-42.

7. Raposo D, Kaufman MT, Churchland AK. A category-free neural population supports evolving demands during decision-making. Nature neuroscience. 2014;17(12):1784-92.

8. Hirokawa J, Vaughan A, Masset P, Ott T, Kepecs A. Frontal cortex neuron types categorically encode single decision variables. Nature. 2019;576(7787):446-51.

9. Grossberg S. Contour enhancement, short term memory, and constancies in reverberating neural networks. Studies in Applied Mathematics. 1973;52:213-57.

10. Wang XJ. Probabilistic decision making by slow reverberation in cortical circuits. Neuron. 2002;36(5):955-68.

11. Standage D, You H, Wang DH, Dorris MC. Gain modulation by an urgency signal controls the speed-accuracy trade-off in a network model of a cortical decision circuit. Front ComputNeurosci. 2011;5:7.

12. Leblois A, Boraud T, Meissner W, Bergman H, Hansel D. Competition between feedback loops underlies normal and pathological dynamics in the basal ganglia. J Neurosci. 2006;26(13):3567-83.

13. Duque J, Lew D, Mazzocchio R, Olivier E, Ivry RB. Evidence for two concurrent inhibitory mechanisms during response preparation. J Neurosci. 2010;30(10):3793-802.

14. Thura D, Cisek P. The Basal Ganglia Do Not Select Reach Targets but Control the Urgency of Commitment. Neuron. 2017;95(5):1160-70 e5.

15. Thura D, Cisek P. A computational model of cortico-basal ganglia circuits for deciding between reaching actions. Society for Neuroscience Abstracts. 2019;Online.

List of legends

Fig A in S1 Text. Schematic diagram of the analysis approach. Step 1: Neural activity is recorded from individual cells while the monkey performs individual trials of different types (e.g., "easy trial to the right", "misleading trial to the left", etc.), and the temporal profile is aligned on movement onset time. Step 2: For each cell, activity is averaged from all trials during the slow block in which the target on the right was chosen, to produce an average "Slow-R all" temporal profile. This is repeated for Slow-L, Fast-R, and Fast-L trial groups. Step 3: The four trial groups are concatenated into a single row for each cell, and the rows of all cells grouped into a large matrix. Step 4: Principal components analysis is performed on that matrix to produce the "loading matrix" L. Step 5: To generate the trajectory for a specific trial type and a specific population, we first average the neural activity of a given cell over all trials of the given type (e.g. misleading trials in the slow block in which the right target was chosen). Step 6: To compute the profile for that trial type and for a given principal component, the average activity of each cell is weighted by the corresponding row (cell) and column (PC) of the loading matrix L, and summed. Step 7: The profile of each PC is divided by the number of cells used. Step 8: The resulting PC profiles are plotted against each other to produce neural trajectories. Here, the trajectory for Slow-Misleading-R trials is highlighted and superimposed over the trajectories of other slow block trials, as in Fig 4A. Note that steps 5-8 can be computed using arbitrary subsets of data (different trial groupings, different subsets of cells), and arbitrary alignment (on movement onset or on the first token jump), because all they depend upon is the loading matrix computed by steps 1-4.

Fig B in S1 Text. The top four principal components produced when the loading matrix was computed from each brain region separately, same format as Fig 2. On the right are shown the neural trajectories computed in the space of the components that reflect commitment, evidence, and elapsing time. Note that in some cases the axis is inverted to facilitate comparison of these neural spaces to those shown in Figs 6 and 7. Data and code available at https://doi.org/10.6084/m9.figshare.20805586.

Fig C in S1 Text. The top four principal components produced when the loading matrix was computed using only 35 cells per region (A), only data from movement onset to 400ms later (B), data without square root transformation (C), only data from the slow block (D), or all data from all 28 trial conditions separately (E). Same format as Fig B. Data and code available at https://doi.org/10.6084/m9.figshare.20805586.

Fig D in S1 Text. Results computed separately for each of the two animals. Panels A and B show the principal components (same format as Fig 2). Panels C and E show the top four components calculated using each brain region (similar to Fig 5). Panels D and F show the decision manifolds for PMd and M1 (similar to Fig 6). Data and code available at https://doi.org/10.6084/m9.figshare.20805586.

Fig E in S1 Text. Results computed without the cell duplication step. A. The top four principal components (similar to Fig 2). B. The neural trajectories and decision manifold for PMd (similar to Fig 6A). C. Neural trajectories and decision manifold for M1 (similar to Fig 2B). D-F. Neural trajectories for dlPFC, GPe, and GPi (similar to Fig 7). G. Loading distributions for PMd and M1 with respect to PC1 and PC2 (similar to Fig 8A and 8C, left). H. Loading distributions for dlPFC, GPe, and GPi with respect to PC1, PC2, and PC4 (similar to Fig 8F). Data and code available at https://doi.org/10.6084/m9.figshare.20805586.

Fig F in S1 Text. Analyses of synthetic populations. A. The top 3 Synthetic PCs (SPCs) obtained from a population of 600 neurons constructed using combinations of the original PCs 1, 2, and 4 (see Fig 2). B. The loading coefficients of all 600 neurons, fitted with GMMs (gray ellipsoids). Colors indicate the three groups of neurons built with different combinations of those original PCs (see legend). C. The top 3 SPCs obtained from a population of 600 neurons constructed only using the original PC1 and PC2, but separated into three distinct categories: cells that are only active during deliberation, cells only active during movement, and cells active during both epochs. D. The loading coefficients of all 600 neurons, fitted with GMMs (gray ellipsoids). Colors indicate the three categories of neurons. Data and code available at https://doi.org/10.6084/m9.figshare.20805586.

Fig G in S1 Text. Simulation of a simple 2-neuron recurrent competitive attractor. A. The structure of the model is shown at left, next to the governing equations and parameter settings. Two forms of the interaction function $f(x)$ are shown in the inset at right. B. The flow field for a system with high gain (G=1) and steep slope (S=15) when the input evidence is balanced such that $E_1=E_2=0.2$. Blue arrows depict the flow and shading indicates the speed (yellow=fastest, dark blue=slowest). The purple outline indicates a region around the stable equilibrium, in which the state will tend to remain even with substantial noise. Note that the dynamics flow quickly toward that region and then move more slowly within it. C. The stable regions of the same system for several input patterns, ranging from strong evidence in favor of choice 2 (top left, blue) to strong evidence in favor of choice 1 (bottom right, red). Note that these align in what is approximately a straight line across the state space (compare to dashed black line). D. Same as B but for a system in which the interaction function has low gain (G=0.1) and a shallow slope (S=5). E. Stable regions of the system in D. Note that now, the stable regions for the same range of input patterns do not form a straight line but rather fall on a curve roughly equivalent to a circle (dashed black curve). F. Same as C and E but for models in which the input itself is constrained to lie on a curve.

Fig H in S1 Text. Comparison of selectivity in GPi with the slope of the PMd decision manifold. A. Comparison within six conditions: all Slow block trials, Slow block trials where DT<1400, Slow block trials where DT>1400, all Fast block trials, Fast block trials where DT<950, Fast block trials where DT>950. Each panel plots the absolute value of PC2 computed from GPi (red, left y-axis, shaded region indicates 95% CI) along with the derivative of PC1 computed from PMd (blue, right y-axis). The black vertical line indicates the estimated time of commitment. At the top left corner are Pearson's R and the p-value of the correlation between these two signals from 1000ms to 280ms before movement. B. The correlation of these signals, from 1000 to 280ms before movement onset, across all 6 conditions. Data and code available at https://doi.org/10.6084/m9.figshare.20805586.
