# Supplementary material for: Integrated neural dynamics of sensorimotor decisions and actions
Source: PLoS Biol. 2022 Dec 15;20(12):e3001861. doi: 10.1371/journal.pbio.3001861 (PMC9754259; doi:10.1371/journal.pbio.3001861)
Supplement: Attachment — Submitted filename: ThuraCabanaFeghalyCisek PLOS_Responses2reviewers_AsSub.docx [file pbio.3001861.s006.docx]

**Responses to reviewers**

We are grateful for the kind comments from both reviewers. We were pleased to hear that they found our article to be important and well written, and that they both support publication in PLOS Biology. Below, reviewers’ comments are in **bold** and our replies in normal font.

***Editorial requests:***

**TITLE:**

**We'd like to suggest a title change that we feel will make this work somewhat more accessible to our broad audience. Something along the lines of:**

**1) Sensorimotor decisions and actions reflect unified neural dynamics**

**2) Movement decisions and actions are mediated by similar neural dynamics**

**3) Movement decisions and actions are mediated by similar neural dynamics in the cerebral cortex and basal ganglia**

Following the spirit of your suggestions, we’ve changed the title to “Integrated neural dynamics of sensorimotor decisions and actions”

**ABSTRACT:**

**Our non-native English speaking editors felt that replacing "implemented" with "coded" or "regulated" or something similar would be preferable in the abstract.**

We’ve carefully considered this suggestion, but respectfully disagree. In our opinion, the word “coded” is highly misused in the field because it carries many implications that are rarely warranted (e.g. that something is later "decoded"), and brings to mind an “information transmission” metaphor that we think is not appropriate for describing sensorimotor processes. "Regulated" is much better, but frankly, we feel that "implemented" is really the best choice, and it is the term used throughout the field to link neural mechanisms to behavioral functions.

Having said that, we’ve changed the last sentence of the abstract to try to be more accessible to a broad audience.

***Reviewer #1***

We thank the reviewer for evaluating our paper and we appreciate her/his enthusiasm and highly supportive comments. We have indeed thought about this data for many years and are gratified that the reviewer finds it to have been worthwhile.

**Given the current writing, it is hard to link the author's underlying hypotheses and the results one would expect. For example, in the first section 'Dimensionality reduction of neural activity into principal components', the authors spend a lot of time discussing how they did PCA and comparative analyses (GPFA). This is fine. But these methodological details seem to be present in place of (1) what the hypothesis being tested is, and (2) what the reader should expect to see in the PCA results given those and alternative hypotheses.**

**In the same 'Dimesionality reduction…' section, the authors bring special focus to PC2. I agree that it is interesting that it shows a split between "easy", "hard" and "ambiguous". But, should we have expected this given the hypothesized overlap between 'choice' and 'execution'? The connections between hypotheses and results are remain somewhat opaque.**

To address the reviewer’s remark on the lack of details regarding the connection between hypotheses and results, we’ve now modified the introduction section (lines 132-138) to clarify our main hypothesis (continuum of selectivity between decision and action-related activities in the population) and state an alternative one (distinct populations of neurons specialized for deliberation versus execution). To illustrate what to expect given this alternative hypothesis, we now refer to earlier work (e.g. Blanchard et al., 2018) and foreshadow the analyses we will perform (Figure 8 and Supplemental Figure S6d).

**It seems useful to use the TME method here. But, it sems like this is being used in place of a (mixed) selectivity analysis. Are we to interpret this control as saying the selectivity is random and mixed across the population? If so, it seems best to show this with something like a pairs or epairs test; note, I realize the authors used a clustering of loading coefficients later.**

**I think part of the claim with TME being made here is that 'no neuron subtype is solely responsible' for these dynamics seen in the PCA. But, how much of these neurons exhibit a pure, linear mixed or nonlinear mixed selectivity? How much of the PCA results are in fact due to dynamic changes in the nonlinear mixtures of units for task-relevant variables (see 5 next, also).**

We used the TME method not to check for mixed selectivity, but to test whether the temporal profiles along PC2, which strongly reflect sensory evidence provided by the jumping tokens during deliberation, are not a simple consequence of cells’ tuning for the left or the right movement. The question of cell selectivity is further addressed below to answer other reviewer’s remarks. Below, we also show results of the PAIRS test suggested by the reviewer.

**If the underlying components of the decision model are a priori known, why is PCA used rather than a focused method such as regression and their underlying subspaces? This would be more theory/hypothesis-driven. If using a regression approach, then the authors could introduce movement execution and choice regressors (and their interaction). Doing this would allow testing the claim they setout to do, but right now, it's unclear what constitutes 'execution' in the PCs; I don't see movement variables, except for left or right. If the authors meant for choice to == integration of evidence, and left/right to == execution, please state this more clearly and why left/right aren't just action rules or space codes.**

We appreciate the suggestions for alternative ways of analyzing our data proposed by the reviewer. However, we feel that the PCA approach is overall well-suited to address our questions. One advantage of using it, as opposed to more targeted methods, is that it is already widely used and well known, relies on very few assumptions, and provides easily interpretable results. Moreover, given its popularity in the field, it makes comparisons between studies very convenient. We have made our data available, along with the code needed to perform our analyses, so readers would be able to apply other methods in the place of PCA. In fact, we would welcome that and be happy to help, perhaps leading to new insights – but that’s beyond the scope of this paper.

The reviewer is right about the direction of movements, which constitutes the only parameter related to movement execution in the PCs. While we agree that other parameters would have been interesting to map, our monkeys’ movements in the tokens task were overall very similar, except for their direction. Movements would thus probably not provide enough variability with respect to other metrics (e.g. speed) so that those metrics would not be clearly reflected in neural trajectories. Thus, on the basis of the present data set we can’t say whether the activity really reflects movements rather than just rules or space. However, decades of work (lines 445-456) have already shown how neural activity in PMd and M1 is related to a variety of more specific movement parameters, including posture, kinematics, and forces. Thus, in light of those studies we believe it is appropriate to interpret our neural activity as related to the specification of movements as opposed to abstract rules or goals.

**Analysis of the loading matrix: I think this analysis has potential to answer some of the authors main questions. However, the section is unclear because the expected results given the hypotheses aren't stated up front. It would help the reader to know what they should be looking for to follow along**

As mentioned above, we hope that we have provided clarification by modifying the introduction section (lines 132-138).

**The authors state (lines 352-353): "this suggests that the lack of distinct clusters….reflect absence of categories in the neural populations". Is this statement meant to convey that the distribution of neuron encoding approaches what many called random mixed selectivity (Raposo et al., 2014; Hirokawa et al., 2019)? If so, I think it would be best to state that clearly and cite those works too.**

We have followed the reviewer’s suggestion to provide more detail on the interpretation of the loading matrix results. We now discuss our results in light of the two studies mentioned by the reviewer (lines 373-383).

Raposo et al. (2014) developed a new statistical technique (projection angle index of response similarity, PAIRS) to identify categories of cells from their selectivity at the population level. They found that the population is category free: response features are randomly distributed across neurons (i.e. no deviations from a random, category-free structure). However, as noted by Raposo et al., the PAIRS test is not only a test of clustering but also a test of the non-uniformity of the population. In their data, the technique revealed both uniformity and lack of clustering. The loading distribution in our data, however, is already known to be non-uniform. For example, the PMd population has a strong tendency for positive loading on PC4 (Figure 8A, rightmost plot), while dlPFC has a tendency for negative loading on PC1 (Figure 8A, leftmost plot). Furthermore, our analyses do show some structure in both PMd and M1 (middle plots in Figure 8A,B). In other words, we already know that the PAIRS test would show a significant departure from a uniform distribution.

Nevertheless, prompted by the reviewer’s advice we did perform the analysis, and an example is shown below (This is analogous to Figure 3e of Raposo et al.):

In blue is the θ­_data_ distribution for PMd and in red the θ­_random_ distribution. As can be seen, the data is significantly shifted to the left, indicating that it is not uniform, as expected. For comparison, here is the same analysis for the synthetic data with deliberate clusters (as in Supplemental Figure 6C,D):

Here, we see an even greater shift, due to the clustering in addition to the non-uniformity. In short, we see the expected results, but that is not really what our analyses were aimed at.

Our analyses were aimed at looking for a more specific type of clustering: whether there is a distinction between cells involved in deliberation versus execution, and we think the GMM analysis is better suited to this type of targeted test (as are related approaches such as those used by Blanchard et al. 2018). The reason is that it looks for clusters in the space of loading coefficients as opposed to just the space of their angles, so it can identify the presence or absence of clusters even if they lie in the same direction in loading space and even if the distribution is non-uniform, as described above. Neither of these properties holds for the PAIRS test or for similar tests that focus on angles such as elliptical PAIRS (ePAIRS) and eRP tests described previously (Cuesta-Albertos et al., 2008; Hirokawa et al., 2019).

Consequently, we have opted to not include these analyses in the paper, and instead discuss the differences between these approaches on lines 373-383.

**abstract: what does "clusters within each region" mean?**

We have modified the sentence with “…but no separate, selective populations of cells within each region.”

**I realize that many of the results come from as much as 7 years ago. As such it is not surprising that many of the references are limited to an earlier time. I do think the paper would be improved by adding many of the important papers that come from the period of more recency.**

**Saxena and Cunningham's paper on the population doctrine should be cited somewhere. Other relevant reviews include Urai and Churchland and Ebitz and Hayden.**

We agree with the reviewer and we have now added citations to several relevant and recent papers in both the introduction (lines 131-137) and the discussion (lines 490-493) sections of the paper, including those mentioned by the referee (Saxena and Cunningham, 2019; Jiang et al., 2020; Russo et al., 2020; Barack and Krakauer, 2021; Ebitz and Hayden, 2021; Saxena et al., 2022; Sun et al., 2022; Urai et al., 2022).

***Reviewer #2***

We thank the reviewer for evaluating our paper. We appreciate her/his very positive option about the study and we were glad to hear that she/he appreciated the unified view that the analyses allowed to reveal.

**Fig. 3 legend, "A. Dotted lines show the sensory evidence […]": There don't appear to be any dotted lines in this panel.**

We’ve changed the lines to dashed, which is easier to see.

**Fig. 4A, B: Is there a way to convince oneself that the final parts of the shown trajectories are related to active movement execution (rather than potentially just passively returning to a "neutral" state for the next trial)?**

This is an interesting possibility that we cannot strictly exclude. As mentioned above in our reply to the other reviewer, the direction of movement was the only movement metric we investigated because direction is the parameter that provides two clearly dissociable groups of movements. Movements are otherwise very similar in terms of other parameters (speed, forces, etc.) that could have been reflected in the final parts of the neural trajectories. However, as mentioned in the manuscript (lines 468-480), this pattern of neural activity reproduces many previous observations from very different tasks, including ones in which monkeys were simply instructed to reach out to a single target. Furthermore, given many decades of research on how movement-time activity in PMd and M1 reflects various parameters of execution, we feel it is likely that these trajectories are also related to those parameters (lines 445-456).

**Lines 377-379, "Importantly, the transition to commitment is not equivalent to the crossing of a neural "threshold", but instead resembles falling off the edge of the decision manifold into an orthogonal subspace that loses sensitivity to evidence.": Why is it not possible to think of reaching this edge as reaching a decision threshold from the point of view of terminating the deliberation phase (ignoring, for the moment, what happens dynamically once the edge is reached)?**

This is an interesting point raised by the reviewer. Strictly speaking, we believe that a neural firing threshold (that could be observed in the activity of individual cells) is different from what looks more like a prolonged transition from one state to the other, defined by the dynamic relationship between population activities. That is, what determines commitment is not a given rate of firing of any given neuron, but rather a relationship between firing rates of many neurons. Another way of saying this is that the “edge” of the decision manifold is oriented obliquely with respect to most neurons’ vector of loading coefficients, and thus will vary across conditions when viewed from the perspective of any given neuron. Finally, we don’t think the edge is so crisp as a quantifiable “threshold”, but rather resembles a gradual transition into a “region of no return”, like a ball rolling off a smoothly rounded hill as opposed to a cliff. We have added some relevant text on line 408.

**"As can be seen […] implying that straightforward summation of the activity of cells tuned to the chosen target may not be constant across trial types, as indeed often observed […].": What about the possibility of a time-variant decision criterion (as proposed in many more recent decision models)? Are the results inconsistent with such an idea?**

No, our results are in fact consistent with a time-variant decision criterion. That is effectively what is produced by a rising “urgency signal”, as many people have suggested.

References

Barack DL, Krakauer JW (2021) Two views on the cognitive brain. Nature Reviews Neuroscience:1–13.

Blanchard TC, Piantadosi ST, Hayden BY (2018) Robust mixture modeling reveals category-free selectivity in reward region neuronal ensembles. J Neurophysiol 119:1305–1318.

Cuesta-Albertos JA, Cuevas A, Fraiman R (2008) On projection-based tests for directional and compositional data. Stat Comput 19:367.

Ebitz RB, Hayden BY (2021) The population doctrine in cognitive neuroscience. Neuron:S0896627321005213.

Hirokawa J, Vaughan A, Masset P, Ott T, Kepecs A (2019) Frontal cortex neuron types categorically encode single decision variables. Nature 576:446–451.

Jiang X, Saggar H, Ryu SI, Shenoy KV, Kao JC (2020) Structure in Neural Activity during Observed and Executed Movements Is Shared at the Neural Population Level, Not in Single Neurons. Cell Rep 32:108006.

Raposo D, Kaufman MT, Churchland AK (2014) A category-free neural population supports evolving demands during decision-making. Nat Neurosci 17:1784–1792.

Russo AA, Khajeh R, Bittner SR, Perkins SM, Cunningham JP, Abbott LF, Churchland MM (2020) Neural Trajectories in the Supplementary Motor Area and Motor Cortex Exhibit Distinct Geometries, Compatible with Different Classes of Computation. Neuron 107:745-758.e6.

Saxena S, Cunningham JP (2019) Towards the neural population doctrine. Current Opinion in Neurobiology 55:103–111.

Saxena S, Russo AA, Cunningham J, Churchland MM (2022) Motor cortex activity across movement speeds is predicted by network-level strategies for generating muscle activity. Elife 11:e67620.

Sun X, O’Shea DJ, Golub MD, Trautmann EM, Vyas S, Ryu SI, Shenoy KV (2022) Cortical preparatory activity indexes learned motor memories. Nature 602:274–279.

Urai AE, Doiron B, Leifer AM, Churchland AK (2022) Large-scale neural recordings call for new insights to link brain and behavior. Nat Neurosci 25:11–19.
